# FUNCTIONAL PERFORMANCE IN THE MODIFIED SHUTTLE TEST IN CHILDREN AND ADOLESCENTS WITH CYSTIC FIBROSIS

**DOI:** 10.1590/1984-0462/2021/39/2019322

**Published:** 2020-08-10

**Authors:** Luanna Rodrigues Leite, Karen Caroline Vasconcelos Queiroz, Cristiane Cenachi Coelho, Alberto Andrade Vergara, Márcio Vinícius Fagundes Donadio, Evanirso da Silva Aquino

**Affiliations:** a Pontifícia Universidade Católica de Minas Gerais - Campus Betim, Betim, MG, Brazil.; b Rede Fundação Hospitalar do Estado de Minas Gerais - FHEMIG, Belo Horizonte, MG, Brazil.; c Pontifícia Universidade Católica do Rio Grande do Sul, Porto Alegra, RS, Brazil.

**Keywords:** Exercise test, Cystic fibrosis, Cardiorespiratory fitness, Respiratory physiological phenomena, Teste de esforço, Fibrose cística, Aptidão cardiorrespiratória, Fenômenos fisiológicos respiratórios

## Abstract

**Objective:**

To evaluate factors associated with the performance of children and adolescents with cystic fibrosis (CF) in the Modified Shuttle Test (MST) and compare it with healthy children and adolescents.

**Methods:**

This is a cross-sectional study, with children and adolescents divided into two groups: cystic fibrosis (CFG) and control (CG). Variables evaluated in the MST: walking distance, test level, heart rate variation (∆Hr), post-test mean arterial pressure (MAP Pt) and peripheral oxygen saturation variation (∆SPO_2_). Statistical analysis included Mann Whitney and Spearman coefficient tests, being significant p<0.05.

**Results:**

Sixty individuals aged 6-16 years old were evaluated. Anthropometric data was similar between groups. Differences between groups were shown for: baseline heart rate (BHr), peak heart rate (PHr), ∆Hr, recovery heart rate (RHr), post-test respiratory rate (PtBr), saturation variables, peripheral oxygen level (SpO_2_B) and level test. The ∆Hr and MAP Pt had a moderate positive correlation with distance and level test for both groups (respectively: r=0.6 / p<0.001; r=0.6 / p<0.001). In CFG, the level test had a significant association (r=0.4 - p=0.02) with %FEV_1_.

**Conclusions:**

Children with cystic fibrosis presented functional limitation in the Modified Shuttle Test, which was influenced by lung function.

## RESUMO

**Objetivo:**

Avaliar os fatores que estão associados ao desempenho de crianças e adolescentes com fibrose cística (FC) no teste modificado de Shuttle (MST) e compará-los com os de crianças e adolescentes saudáveis.

**Métodos:**

Estudo de corte transversal com crianças e adolescentes divididos em dois grupos: grupo controle (GC) e grupo FC (GFC). As variáveis avaliadas no MST foram: distância caminhada, nível do teste, variação da frequência cardíaca (∆Fc), pressão arterial média pós-teste (PAMPt) e variação da saturação periférica de oxigênio (∆SpO_2_). Na análise dos dados, foram utilizados o Teste Mann-Whitney e o coeficiente de Spearman, sendo significante p<0,05.

**Resultados:**

Avaliaram-se 60 indivíduos (6-16 anos). Os grupos foram homogêneos em relação aos dados antropométricos. Foi observada diferença significante na frequência cardíaca basal (FcB), na frequência cardíaca de pico (FcP), na ∆Fc, na frequência cardíaca de recuperação (FcR), na frequência respiratória pós-teste (FRPt), na saturação periférica de oxigênio basal (SpO_2_B) e no nível do teste. A ∆Fc e a PAMPt tiveram correlação moderada positiva (respectivamente, r=0,6 / p<0,001; r=0,6 / p<0,001) com a distância caminhada e o nível do teste em ambos os grupos. No GFC o nível do teste teve associação (r=0,4 / p=0,02) com a porcentagem do predito do volume expiratório forçado do primeiro segundo (%VEF_1_).

**Conclusões:**

Crianças e adolescentes com FC apresentaram limitação funcional no teste modificado de Shuttle, influenciada pela função pulmonar.

## INTRODUCTION

Cystic fibrosis (CF) is a genetic condition with autosomal recessive inheritance pattern and systemic onset.^1^ Its main complications involve gastrointestinal alterations, pancreatic insufficiency and severe lung infection, which affects more than 95% of the patients.^1^ ^,^ ^2^ Pulmonary complications usually determine the final prognosis of the disease.^1^ The main consequences of the systemic effects of CF are related to intolerance to exercise,^3^ and different aspects such as malnutrition, ventricular dysfunction, limited air flow, deconditioning, and hypoxemia, which collaborate with the limited exercise capacity in these individuals.^4^

The evaluation of the functional capacity of the patients with CF is efficient to define the prognosis and measure the effects of the disease on activities of daily living. The exercise capacity can be assessed through laboratory and field tests, such as the modified shuttle test (MST) ^5^, which has 15 levels. Participants must walk (or run). The original protocol of this test contains 12 levels and was described by Singh et al.^6^ The MST behaves as a maximum test for most patients, and allows the examiner to assess the physiological response of the individual with CF during the exercise.^7^ ^,^ ^8^ The overload imposed to the cardiorespiratory system during the application of this test may show physiopathological changes related to the condition, which are not identifiable in pulmonary function tests.^9^

Several studies were conducted with the Shuttle test with 12 levels to follow up the population with CF;^7^ ^,^ ^8^ ^,^ ^10^ however, there are few studies assessing which factors are related to cardiorespiratory performance in the MST and which of them may be connected with worse performance in patients with CF. Theferore, based on this information, this study aimed at comparing the cardiorespiratory overload among children and adolescents with CF and healthy individuals, besides evaluating the factors associated with the performance in the MST.

## METHOD

Cross-sectional study approved by the Research Ethics Committee of Pontifícia Universidade Católica de Minas Gerais (Certificate of Presentation for Ethical Consideration 54142716.8.3001.5119) and by the Committee of Fundação Hospitalar do Estado de Minas Gerais (FHEMIG) (1.753.013).

Children and adolescents with CF who did not present with pulmonary exacerbation were selected and followed up at Hospital Infantil João Paulo II (HIJPII), in FHEMIG, in Belo Horizonte, besides healthy children and adolescents attending public schools. Participants were paired according to sex and age. The study began after the consent and assent forms were signed by tutors and participants, respectively. The exclusion criteria were considered in the exercise test for both groups: hemodynamic instability, significant changes in heart rate and blood pressure, orthopedic, neurological or rheumatologic disorders. In the group of healthy children, those who presented with respiratory disorders, pointed out by the International Study of Asthma and Allergies in Childhood (ISAAC), were excluded. ISAAC is validated, reproducible and easy to apply, translated to Brazilian Portuguese.^11^

Two variables of response were defined: walking distance, test level, and both refer to functional performance. The following were mentioned as co-variables: heart rate variation (∆Hr), post-test mean blood pressure (Pt MBP) and peripheral oxygen saturation (∆SpO_2_).

Pulmonary function was assessed in both groups, according to the guidelines of the American Thoracic Society (ATS).^12^ Forced expiratory volume in the first second (FEV_1_), forced vital capacity (FVC) and FEV_1/_FVC were the studied variables, obtained according to the maximal expiratory flow-volume curves using a Jaeger FlowPro spirometer (Erich Jaeger GmbH, Germany). The results were presented in percentage numbers, according to the equations of reference.^13^

The adapted protocol of 15 levels of the Shuttle test, described by Bradley et al.,^14^ was used according to the guidelines proposed by the ATS and by the European Respiratory Society.^15^ The test was carried out in a 10 m corridor limited by cones. The children and adolescents were advised to walk or run, maintaining the speed imposed by beeps. At the beginning of the walk, speed was 0.5 m/s, increasing 0.17 m/s per minute. The increasing velocity was established by a triple beep. The criteria to interrupt the test were: the individual reaching the maximum heart rate (Hr), not being able to complete the route in the delimited time twice in a row, having intense dyspnea, presenting with chest or leg pain, paleness, presenting oxygen saturation lower than 85%. Peripheral oxygen saturation (SpO_2_) and Hr were constantly measured during the test, and the respiratory rate (Rr), blood pressure (BP) and modified Borg scale^16^ were assessed before and after the test. Two MSTs were applied for each participant, with at least 30 minutes of interval between them, as long as the vital data returned to basal values. The longest distance, that is, the best test, was considered for analysis. In case it had been necessary, oxygen would have been offered, according to recommendations.^17^ Both tests were accompanied and conducted by the same evaluator.^10^ At the end of each level, volunteers were told to go a bit faster.^14^

During the test, the verification of Hr was carried out with a heart sensor, which sent the data to a wrist watch (Polar Electro Oy, Model 90440, Kempele, Finland). SpO_2_ was measured by pulse oximetry (Nonin Medical, INC Model 9500 Finger Pulse Oximeter, United States).

Sample was calculated to compare the groups using the software GPower 3.1, which considered power of 90%, alpha error of 0.05 and effective size of 0.80. Thus, sample size in each group was estimated in 28 subjects.^18^

Statistical analysis was performed using the Statistical Package for the Social Sciences (SPSS), version 22.0. The normality of data was assessed by the Shapiro-Wilk test, and demonstrated asymmetric distribution. Therefore, data were expressed in median and interquartile range, and non-parametric tests were used. The Mann-Whitney test was used for the comparison between groups. For the correlation of MST performance variables and co-variables, the Spearman coefficient was used. N all analyses, the considered significance was 0.05.

## RESULTS

The evaluation included sixty individuals distributed in two groups, homogeneously: control group (CG) and CF group (CFG). The median age of participants was 10.5 years, of which 60% were male. In the CFG, 23.5% were classified as eutrophic; 60%, malnourished (low weight); and 16.5% were overweight. In the CG, 30% were eutrophic; 43.3% presented with low weight; and 26.7% were overweight. There was no statistical difference between the assessed groups regarding anthropometric data. In CFG, the most common genetic mutation and bacterial colonization were: 508del heterozygote, in 53%, and *Staphylococcus aureus*, in 70% of the patients (Table 1). Lung disease in the individuals was classified according to FEV_1_, as follows:

- 33.5%: mild.
- 30%: moderate.
- 10%: severe.
- 26,5%: did not present with lung disease.^8^

**Table 1:** Genetic and respiratory conditions of the cystic fibrosis group.

n	Genetics	Colonization	Pulmonary function		
			FVC%	FEV_1_%	FEV_1_/FVC%
1	508del, 508del	*Staphylococcus aureus*	71.3	65	99.9
2	508del, 508del	*Staphylococcus aureus*	65.6	46.2	77.1
3	R1162X, 508del	*Staphylococcus aureus*	63.6	47.6	82.3
4	508del, 508del	*Staphylococcus aureus*	104	97.4	93.3
5	G85E, 508del	*Staphylococcus aureus*	95.8	89.2	97.5
6	508del, 508del	*Staphylococcus aureus*	65.6	46.2	77.1
7	508del, c.695T>A	*Staphylococcus aureus*	92.5	82.2	76.75
8	N1303K,508del	*Staphylococcus aureus*	92.6	80.8	96.6
9	p.lle506del, unidentified heterozygote*	*Staphylococcus aureus*	84.7	69.8	91.6
10	c.1116+1G>A, 508del	*Pseudomonas aeruginosa*	61.9	49	85.9
11	508del, 1624G>T	*Haemophilus influenzae*	124.4	113.2	99.2
12	c.695T>A, c3745G>C	*Staphylococcus aureus*	111.6	95.1	89.6
13	508del, 508del	*Pseudomonas aeruginosa*	93.2	88.3	102.1
14	1248+1G>A, 508del	*Staphylococcus aureus*	99.3	90.6	100
15	508del, 508del	*Staphylococcus aureus*	89.0	82.2	97.1
16	c.1624G>T, c.1680-1G>A	*Pseudomonas aeruginosa*	33.7	31.6	93
17	508del, c3484C>T	*Pseudomonas aeruginosa*	58.2	45.6	84.8
18	508del,3120+1GA	*Staphylococcus aureus*	83	69.7	89.2
19	508del, 2184insA	*Pseudomonas aeruginosa*	76.2	55.4	80.2
20	508del, c.2051_2052	*Staphylococcus aureus*	98.3	88.4	96.7
21	508del, c.11C>A	*Pseudomonas aeruginosa mucoide*	49.9	34.1	70.5
22	508del, G85E	*Haemophilus influenzae*	78.7	74.2	100.5
23	508del, R1066C	*Staphylococcus aureus*	72.2	60.3	88.4
24	508del, 3120+1G>A	*Staphylococcus aureus*	90.9	76.6	84
25	R1066C, 508del	*Staphylococcus aureus*	76.5	71.3	102.74
26	508del, c.4124A>C	*Staphylococcus aureus*	102	89.9	92.8
27	508del, c.3717G>A	*Staphylococcus aureus*	135.9	111.0	81.9
28	508del, 508del	*Staphylococcus aureus*	69.3	73.4	110.4
29	508del, c.1682C>A	*Staphylococcus aureus*	90.3	90.1	97.9
30	508del, 508del	*MRSA*	44.7	23.3	55

n: number of individuals; FVC%: percentage of the forced vital capacity predictor; FEV_1_%: percentage of the forced expiratory volume in the first second predictor; FEV_1_/FVC%: percentage of the Tiffeneau index predictor; *two sweat tests changed with chloride ions >60 mmol/L; MRSA: methicillin-resistant *Staphylococcus aureus*.

**Figure 1:**
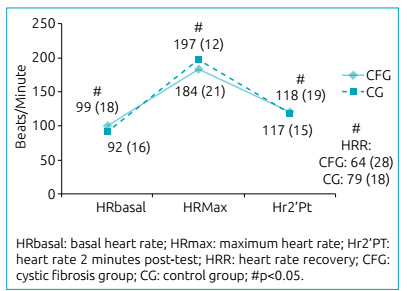
Heart rate behavior in the Modified Shuttle Test.

The Hr behavior in the test is presented in Figure 1. This variable showed similar linear behavior in both groups; however, basal heart rate (HRbasal) was higher in the CFG, and maximum heart rate (HRmax) was higher in the CG. Heart rate recovery (HRR) was shown by the difference HRmax by the heart rate two minutes after the test (Hr2’Pt)

**Figure 2:**
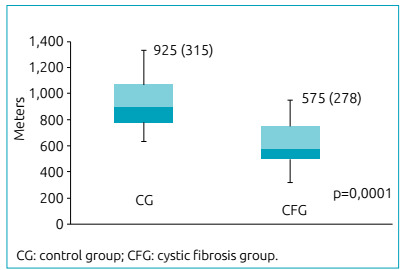
Walked distance: control group and cystic fibrosis group.

In the comparison between groups, there were statistically significant differences in the variables HRbasal, HRmax, ∆Hr, HRR, post-test respiratory rate (PtRR), peripheral basal oxygen saturation (SpO_2_B) and advanced level (Table 2). The CG presented cardiac overload7% higher in comparison to the CFG. The distance walked or run also indicated statistical difference between groups (Figure 2) (p=0.0001). The CFG showed a 35% reduction in the walked distance in comparison to the CG.

**Table 2:** Comparison of the variables in the Modified Shuttle test between the control group and the cystic fibrosis group.

	CG	CFG	p-value
	Median (interquartile range)	Median (interquartile range)	
HRbasal	92 (16)	99 (18)	0.035
HRmax	196 (11)	183.5 (21)	<0.001
∆Hr	101.5 (19)	79 (28)	<0.001
HRR	79 (18)	64 (28)	0.04
Pt RF	44 (8)	40 (9)	0.001
Pt MBP	80 (20)	80 (10)	0.806
∆SpO_2_	-0.5 (2)	-2 (7)	0.035
BorgPt	8 (6)	5 (5)	0.577
Level	12 (2)	9 (2)	<0.001

CG: control group; CFG: cystic fibrosis group; HRbasal: basal heart rate; HRMax: maximum heart rate; ∆Hr: heart rate variation; HRR: heart rate recovery; Pt RF: post-test respiratory frequency; Pt MBP: post-test mean blood pressure; ∆SPO_2_: peripheral O2 saturation variation; BorgPt: Borg post-test.

The correlations between the performance variables and the studied co-variables are in Table 3. We observed that ∆Hr and Pt MBP, respectively, had positive and significant moderate correlation with distance (r=0.6 and p<0.001; r=0.6 and p<0.001) and test level (r=0.6 and p<0.001; r=0.6 and p<0.001) in both groups. In the two analyzed groups, Pt MBP increased when individuals reached longer distances and levels in the test. The better the functional performance, the higher the cardiorespiratory overload imposed to the individuals. The ∆SpO_2_ presented moderate and significant correlation with the walked distance in the two assessed groups. The longer the distance reached by the CFG, the higher the saturation variation (r=0.4; p=0.04). In the CG, the lower the saturation variation in the test, the higher the functional performance (r=-0.4; p=0.04).

**Table 3:** Factors associated with performance in the Modified Shuttle Test.

	CG (n=30)				CFG (n=30)			
	Level		Distance		Level		Distance	
	*r*	p-value	*r*	p-value	*r*	p-value	*r*	p-value
∆Ht	0.6	<0.001	0.6	<0.001	0.6	<0.001	0.6	<0.001
Pt MBP	0.6	0.001	0.6	<0.001	0.6	0.001	0.6	<0.001
∆SpO_2_	-0.3	0.115	-0.4	0.04	-0.3	0.115	0.4	0.04

CG: control group; CFG: cystic fibrosis group; ∆Fc: heart rate variation, Pt MBP: post-test mean blood pressure; ∆SpO_2_: peripheral O2 saturation variation; r: Spearman correlation.

In both groups, the association between the performance variables and the pulmonary function of participants was also assessed. The reached level presented moderate positive correlations with the percentage of the FEV_1_ predictor in the CFG and the CG (r=0.4 and p=0.02; r=0.5 and p=0.00), respectively. The correlations between the percentage of the forced vital capacity (%FVC) predictor and the response variables in both groups were moderate, positive and significant, both for distance (CFG and CG: r=0.4 and p=0.02) and reached level (CFG: r=0.4 and p=0.03; CG: r=0.4 and p=0.02).

## DISCUSSION

The main findings of this study showed that the factors associated with the best performance in the test were ∆H_r_, Pt MBP and ∆SpO_2_ in both groups. Besides, int he CFG pulmonary function was closely related to the performance in the test. The impact of lung disease relates to the functional worsening in the MST of children with CF, resulting in a shorter distance when compared to healthy individuals.

This study pointed out that MST caused the Hr to increase in both groups. We applied the Shuttle test with 15 levels, in which the participant can run, besides walking. This fact may have contributed with the higher increase in Hr, as observed by Lanza et al.^10^ Therefore, another study^5^ evaluated adolescents with CF using the 6-minute walk test (6 MWT) and MST, and concluded that, in the MST, the cardiac overload was higher in comparison to the 6 MWT; therefore, this test is a good option for planning interventions and programs of reconditioning and rehabilitation for children and adolescents with CF.

One criterion addressed at assessing the physiological responses of the cardiopulmonary system to exercise is Hr behavior^5^ ^,^ ^19^ According to the results, there was higher cardiac overload and better performance in the CG test. Therefore, ∆Hr was higher in healthy individuals. Besides, the population with CF presented with higher HRbasal and lower HRR in comparison to the CG.

Florêncio et al.^20^ studied ∆Hr in 25 children and adolescents with CF and compared their results to those of healthy children. In their research, they used the 6 MWT and verified lower heart rate recovery rate in the CFG than in the CG. Besides, individuals with CF obtained higher HRbasal. The justification for that, according to the authors, is that individuals with CF presented with higher sympathetic drive, besides changing in breathing patterns, higher respiratory demand and increased circulating catecholamines.^20^ Oliveira and Santos^21^ mention that the increased energy metabolism at rest leads these patients to increase their Hr because of inflammatory processes caused by the increased circulation of pro-inflammatory cytokines.

Ketchell et al.^22^ developed a large longitudinal study with 146 patients who were in the lung transplant waiting list. These patients were assessed with the Shuttle test with 12 minutes and the 6 MWT. Mortality was higher in the group in which patients’ HRbasal was higher than 120 bpm, assessed in the Shuttle test. These patients presented risk of death in up to 500 days after the evaluation in the test. Another important finding is that the risk of death reduced with time, when patients presented with lower HRbasal.^22^

Efficient Hr recovery is considered as a marker of physical aptitude and favorable prognosis in the follow-up of patients with CF.^23^ ^,^ ^24^ A study carried out with 2,193 patients in the United States showed that HRR lower than 22 bpm two minutes after stopping the exercise is related to a higher risk of death among individuals with CF.^24^ Therefore, HRR was assessed both in the CG and in the CFG in the second minute post-test. The findings in this study show that both groups presented favorable Hr post-test recovery; however, individuals with CF had lower recovery in comparison to the healthy ones.

Mean BP is an indirect measurement that includes systolic and diastolic BP. The BP of children and adolescents behaves similarly to that of adults; values are proportional to age and intensity of the exercise.^25^ This study showed that Pt MBP was directly associated with the functional performance in the test in both groups. A Brazilian study associated the increasing systolic BP with the increasing intensity of the exercise.^25^ This study had a similar result, once the increasing walked distance and the intensity of the test led to increased Pt MBP.

Regarding SpO_2_, this variable also presented an association with performance in both groups. In the CFG, the better the performance assessed using the walked distance, the higher the oxygen desaturation, which demonstrates that the overload imposed to the respiratory system had a direct interference on saturation reduction. The O_2_ desaturation in exercise in the population with CF may occur because of the damaged pulmonary function triggered by the condition. The increased effort requires higher ventilatory response; however, because of the compromised lung capacity, the gas exchange is inefficient, so there is incompatibility in the ventilation-perfusion ratio, and reduction in gas diffusion.^26^ Vallier et al.^27^ compared the physiological responses of the cardiopulmonary exercise test (stationary bicycle) with MST and concluded that the Shuttle test is the best choice to detect a reduction in SpO_2_ during exercise in patients with CF. This can be explained because this test represents the activities of daily living, and more muscle mass involved in walking or running. Other authors mention that Sp O_2_ is an important variable to determine the clinical condition of the patient with CF, because the Hr and SpO_2_ response is associated with the severity of the disease.^17^

Concerning the walked distance, the CFG presented with worse performance in comparison to the CG. This situation may have been owed to the limitation of exercise, which is common in this population.^6^ ^,^ ^7^ ^,^ ^26^ In this study, the results of walked distance, both for the CFG and the CG, showed similar behavior to that found in the study conducted by Bladley^8^, who applied the same testing protocol.

The reduction in pulmonary function is one of the main consequences of the comorbidities caused by CF. In this study, we observed that in the CFG, the higher the values of FEV1 and FVC, the higher the walked distance in the MST. The reduced pulmonary function had a direct impact on functional ability, assessed with the MST. Previous studies with the population of patients with CF showed similar results.^6^ Doeleman et al.^28^ analyzed 127 individuals with CF through pulmonary function and MST and found similar results, once they pointed out that the 1% reduction in pulmonary function had an impact of less 15 meters in the walked distance during the test. Klijn et al.^29^ confirm this finding, since they report that the performance in endurance training of patients with CF is related to the reduced pulmonary function and nutritional status, which directly affects the walked distance.

The proper weight and normal muscle mass are directly related to adequate growth and good pulmonary function in patients with CF. Lung disease can often be related to nutritional decline.^30^ In spite of that, this study found no differences between the anthropometric variables of the CG and the CFG. This may have occurred because there is a direct association between the nutritional factor with the pulmonary function test. Here, 60% of the individuals with CF were classified with a mild disorder or no respiratory disorder. These results are in accordance with the findings of Florêncio et al.,^20^ who analyzed a CFG with mild lung disease and did not find differences in the anthropometric data between healthy children and those with CF.

The level of pulmonary dysfunction in patients with CF is a limitation of this study, since 60% of them had mild or no dysfunction. Therefore, a population whose profile was stratified by the level of lung dysfunction could have increased the inference of the associations. Besides, due to its cross-sectional design, the study does not allow the establishment of a cause-and-effect ratio; for that, a longitudinal cohort should be analyzed.

The conclusion was that patients with CF present with higher functional and cardiac limitation, assessed by the MST, in comparison to healthy individuals, and that factors associated with performance were similar in both groups. The anthropometric variables did not interfere in the performance of the groups, and cardiopulmonary function directly influence the functional capacity of individuals with CF.
